# Examining key factors impact on health science students’ intentions to adopt genetic and pharmacogenomics testing: a comparative path analysis in two different healthcare settings

**DOI:** 10.1186/s40246-022-00382-3

**Published:** 2022-03-14

**Authors:** Margarita-Ioanna Koufaki, Stavroula Siamoglou, George P. Patrinos, Konstantinos Vasileiou

**Affiliations:** 1 Department of Pharmacy, Laboratory of Pharmacogenomics and Individualized Therapy, University of Patras School of Health Sciences, University Campus, Rion, 265 04 Patras, Greece; 2 College of Medicine and Health Sciences, Department of Genetics and Genomics, United Arab Emirates University, Al-Ain, United Arab Emirates; 3 Zayed Center for Health Sciences, United Arab Emirates University, Al-Ain, United Arab Emirates

**Keywords:** Path analysis, Comparative analysis, Questionnaire survey, Genomics, Genetic testing, Health science students, Perceptions, Intentions to adopt, Different cultural settings

## Abstract

**Background:**

There is an increasing interest worldwide in investigating healthcare stakeholders’ perceptions and intentions to adopt pharmacogenomics (PGx) into clinical practice. However, the existing inquiries based on well-established theories and models that interpret their intentions to implement PGx are scarce. This study is the first that examines the impact of selected factors on health science students’ intention to adopt genetic testing applications using the technology acceptance model while it compares two different cultural groups: Greeks (Europe; Christian) and Malays (Asia; Muslim).

**Results:**

Malay students were more persuaded about benefits of genomics for drug management compared to their Greek counterparts. However, participants from both countries appear to be particularly convinced about the benefits of genomics on disease management. Moreover, students from both countries considered the potential misuse of genetic information by corporate or government bodies as their most important concern; Greek students appeared to be considerably less worried than Malay about other probable hazards such as the deficient protection of privacy and confidentiality, which could be attributed to their religious background. Participants from both samples expressed very positive attitudes towards genetic research and testing and their favourable intentions to adopt genetic testing for personal use. Exploratory factors analysis and path analysis yielded quite similar results for both samples. Path analysis revealed that the factors of attitudes, concerns, drug management benefits and disease management benefits significantly influenced students’ intentions to adopt genetic testing for personal use, with attitudes being the most inspirational factor with rather high impact, while training did not seem to affect participant’s intentions. The squared multiple correlation of both models was quite satisfactory reaching to 0.55 for the Malaysian sample.

**Conclusion:**

Similarities in the results of the two groups along with the relevant validity and reliability tests indicate that the proposed model is a good fit for future studies to interpret stakeholders’ intentions to adopt genetic testing. Therefore, it can provide a promising and reliable basis for future model development to explain the relationships between intentions to adopt genetic testing and its predictors.

**Supplementary Information:**

The online version contains supplementary material available at 10.1186/s40246-022-00382-3.

## Introduction

During the past decades, genetic research has played an essential role in understanding diseases’ pathophysiology and patients’ input, underscoring the impact of personalized medicine (PM). PM as a clinical approach has a beneficial impact on patients, health care delivery systems and researchers by offering adequate therapeutic approaches to each patient and saving time, effort, and money for disease and drug management [1].

Nowadays, PM significance and contribution in clinical practice has been acknowledged, and for this reason, there is a plethora of research studies available in the literature. The majority of these studies aim to explore and understand the attitudes and perceptions of healthcare professionals, health science students and patients towards the use of genetic testing in clinical practice, the benefits and risks of genetic applications such as pharmacogenomics (PGx) testing and the potential barriers that impede their wider implementation. Admittedly, the adoption level of genetic testing varies across the globe [2]. In western societies, for instance, the genetic testing applications such as pharmacogenomics (PGx) in clinical practice are popular, whereas in Asia, PGx implementation is limited and restricted only in urban areas [3–5].

Notably discrepancies may be attributed to the existence of different cultural backgrounds, professionals' perceptions, society ethnicity mix and the way the health system works which is of major importance [2, 6]. Even if there is a great number of publications addressing the possible barriers that impede PM implementation and the use of genetic testing, there are only a few studies focusing on determining and empirically investigating the factors and their relationship to the adoption of genetic testing. This gap in the literature is significantly important since understanding the way these factors affect the rate of adoption and recognizing the influence of cultural differences in the implementation of innovative services is essential to develop and enact more targeted healthcare policies [7, 8, 9].

Our group has an active research interest in assessing and reporting the level of awareness of pharmacy students regarding genetic testing and its application in Greece [8]. In previous surveys, we have investigated the perceptions and attitudes of medical and pharmacy health science students towards genetic testing in Southeast Asia and Southern Europe, gaining insight into their existing perceptions [10, 11]. Evidently, it was possible to estimate the adoption level of PM in Malaysia and Greece correspondingly by pinpointing the main factors that affect genetic testing adoption and to explore the impact that cultural differences exert on technological innovation acceptance.

Here, we suggest a new model which investigates the impact of selected factors on health science students' intention to adopt genetic testing by probing into the similarities and differences of two disparate cultural settings, namely a European, Greece, and an Asian, Malaysia.

## Methods

### Framework for the proposed model

In this study, we have developed a customized model framework to estimate the impact of several factors on health science students’ intention to adopt a new innovation such as genetic testing using technology acceptance model (TAM). TAM is a behavioural model which intends to discover and highlight the principles and mechanisms of human conduct and to determine the factors which formulate people’s perceptions and attitudes towards technology acceptance and adoption of innovative products or services. In terms of healthcare innovation, scientists prefer TAM because it is dedicated to study user acceptance and use of health technology by examining the effect of attitudes, intentions and social rules in peoples’ behaviour [12]. This information system theory is able to model the way users accept and make use of a new technological advance. By combining two variables notably perceived usefulness (PU) and perceived ease of use (PEU) of the technology, TAM manages to find out the factors affecting a person’s decision related to how and when a person will use a technology [13]. In addition, it is an easily adjustable model to meet the objectives of each study, a feature that makes it more suitable for healthcare innovation [12].

As mentioned before, this study aims to investigate the health science students’ intentions to adopt genetic testing applications in two different cultural backgrounds. In general, the literature so far shows a great effort in understanding the level of awareness of healthcare professionals. Indeed, respondents in several studies stated that the implementation of genetic testing has a positive impact in drug management because it supports the delivery of personalised pharmacotherapy services. By adjusting the adequate dosage scheme to each patient’s needs, it manages to reduce the incidence rate of adverse drug reactions and the expenditures for medications [14–16]. In addition, when students were asked about how genetic testing can influence their health, most of them believed that it could moderately influence it, highlighting the importance of relevant testing in disease management thanks to its contribution in prevention, early diagnosis and treatment [17]. Apart from the benefits of genetic testing, studies have highlighted the existing risks and concerns that pose a burden in its adoption. Most healthcare professionals are significantly worried about the shortage of available resources to apply genetic testing and its various applications, and about the lack of clinical guidelines, while they pounded the alarm about confidentiality and discrimination issues that may occur due to the improper usage and storage of data [18, 19]. Furthermore, the level of knowledge, expertise and genetic training of professionals in the field was found to be low or insufficient to support such innovations [20–22]. Despite the low level of knowledge among healthcare professionals, it is noticeable that there is a rather positive attitude towards the adoption of genetic testing and of PGx in particular, in the clinical setting [16, 23]. Finally, other barriers impeding genetic testing implementation are religious matters and the potential psychological impact that such a test may provoke [24].

Nonetheless, there are only a few research projects that apply TAM or other related theories, and they are dedicated to unveiling the factors which determine healthcare professionals’ intention to adopt genetic testing in their everyday life [14, 25]. According to those, factors such as perceived benefits from the innovation, subjective norms, self-efficacy or self-use, concerns and level of knowledge and training are the most common factors that influence a person’s intention to adopt a technology [26, 27].

**Fig. 1:**
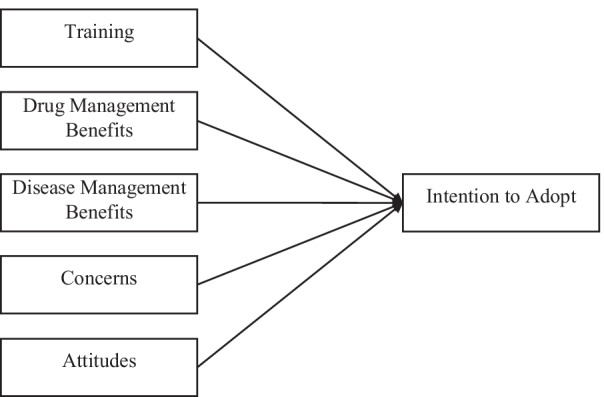
Theoretical model of students’ intention to adopt genetic testing and its predicting factors

Based on the aforementioned findings derived from an extensive literature review, we chose to include the following variables: genomic training, benefits of genetic testing on disease management, benefits of genetic testing applications on drug management, concerns and attitudes (Fig. 1) as factors that can have an impact on a person’s intention to adopt PGx.

### Survey instrument

The instrument employed in this research was a validated questionnaire elaborated by the Laboratory of Pharmacogenomics and Individualized Therapy at the Department of Pharmacy, University of Patras, Greece in collaboration with the Department of Social and Preventive Medicine, University of Malaya, Malaysia which has already been published [10, 11]. Considering its aim and objectives, this study focused on students’ perceptions probed by close-ended questions regarding 6 topics of interest; (1) Students’ genetic training, (2) Benefits of genetic testing on disease management, (3) PGx benefits on drug management, (4) Concerns (risks) about genetics, (5) Attitudes towards genetics’ research and testing usefulness and (6) Students’ intention to adopt genetic testing for personal use (Additional file 1: Table S1). A 5-point Likert scale ranging from “strongly disagree” (= 1) to “strongly agree” (= 5) was deployed for these questionnaire items. Additionally, student demographics such as gender, study course, year of course were taken into account. The study was reviewed and approved by the Institutional Review Board of the University of Patras.

### Study sample

The survey sample consisted of health science students from Greece and Malaysia, a Southeast European and a Southeast Asian country, respectively. Specifically, 205 students from the Health Sciences School of the University of Patras, Greece, and 201 Pharmacy and Medical students from the University of Malaya, Kuala Lumpur, Malaysia, participated in the study (Table 1) and were asked in person to complete a self-administered questionnaire during May 2018 and October–November 2017, respectively. Student samples presented similarities in terms of gender and study course. In both cases, female students outnumbered male counterparts (63.4% in Greece, 72.6% in Malaysian), while around 60% of the participants were medical students. In addition, the Greek sample consisted to great extent, of first year students (around 84%) in contrast to the Malay sample in which participants were more evenly distributed regarding their year of study, and about half of them attended the second year and nearly 40% the third year, while the rest of 7.5% the fourth and fifth year. Therefore, the Malay students involved in the survey are expected to be more familiar with the topic and they have attended more courses relevant to human genomics, genetics and PM than their Greek counterparts.

**Table 1:** Sample descriptive statistics (valid %)

Variables	Greece (*N* = 205)		Malaysia (*N* = 201)	
	*N*	%	*N*	%
*Gender*				
Male	75	36.6	55	27.4
Female	130	63.4	146	72.6
*Study year*				
1	172	83.9	1	0.5
2	32	15.6	107	53.2
3	0	0	78	38.8
4	1	0.5	11	5.5
5	0	0	4	2.0
*Department*				
Pharmacy	77	37.6	82	40.8
Medicine	128	62.4	119	59.2

### Data analysis

Study data were initially analysed using SPSS statistical program (version 27; IBM, NY, USA). Data analysis included frequencies and percentage of valid responses (valid %), descriptive statistics [mean value, standard deviation (SD)]. Moreover, exploratory factor analysis, accompanied with relevant reliability and validity tests [e.g. Cronbach’s alpha, average variance explained (AVE), composite reliability (CR)], was chosen to be conducted thanks to their accuracy to reveal the latent structure of the observed variables. Mann–Whitney 2-Independent Samples test was utilized to specify the scale of differences between the Greek and Malay samples concerning the questionnaires’ answers. Finally, path analysis was run using AMOS (version 26; IBM, NY, USA) to examine the relationships among the factors accrued by the factor analysis and the student intention to adopt genetic testing.

## Results

### Exploratory factor analysis

All aspects of exploratory factor analysis used to confirm the validity and reliability of the results, indicated that the sample and the questionnaire is well-suited and valid. Indeed, FA (Extraction Method: Principal Component Analysis; Rotation Method: Varimax with Kaiser Normalization) for both samples revealed five factors which are expected to affect students’ intention to adopt a genetic testing (Table 2). Bartlett’s test of sphericity yielded a statistically significant value (Chi-Square = 1261.43, *p* = 0.000 for Greece; Chi-Square = 1980.78, *p* = 0.000 for Malaysia) suggesting that there are substantial (overall significantly different from zero) correlations between variables. Moreover, Kaiser–Meyer–Olkin (KMO) measure of sampling adequacy was well above the critical value of 0.5 (0.701 and 0.848, respectively, for Greece and Malaysia) indicating the presence of a strong partial correlation among variables. These indices implied that the matrix was well suited for FA. Moreover, the percentage of variance was 56.65% and 65.85%, for the Greek and Malaysian groups, respectively, while Cronbach’s alpha coefficients for the Greek sample ranged from 0.68 to 0.82 and from 0.73 to 0.89 for the Malaysian, indicating a rather strong internal consistency and reliability among different measurements using same factors. Furthermore, AVE values for both the Greek (ranging from 0.46 to 0.71) and the Malaysian group (ranging from 0.53 to 0.75) were close to the recommended value (≥ 0.5), a fact that indicated a convergent validity. The square of the correlation among all investigated factors was zero in all cases, a finding that confirmed the factors’ discriminant validity for both samples compared to the AVE values. CR values of all factors for both samples (ranging from 0.77 to 0.89) are well above the critical value of 0.7, implying the factors’ internal consistency. Evidently, it was proven that all reliability tests performed corroborated that both models were good fit.

**Table 2:** Results of exploratory factor analysis

Factors and variables	Greece (*N* = 205)					Malaysia (*N* = 201)				
	Factor loadings (56.65%)					Factor loadings (65.85%)				
	Fac. 1	Fac. 2	Fac. 3	Fac. 4	Fac. 5	Fact. 1	Fac. 2	Fac. 3	Fac. 4	Fac. 5
*Genetics training*										
Draw a pedigree					0.841					0.871
Discuss with a family the results of a genetic test and consult					0.847					0.861
*Benefits on disease management*										
Diagnosis				0.720				0.779		
Treatments				0.696				0.813		
Prevention				0.693				0.723		
Prognosis				0.587				0.716		
*Benefits on drug management*										
Drug efficacy increase		0.627				0.694				
Medication cost reduction		0.711				0.755				
Incidence of adverse drug reactions reduction		0.786				0.854				
Severity of adverse drug reactions reduction		0.765				0.849				
Exacerbation reduction		0.623				0.784				
*Concerns (risk) about genetics*										
Privacy and confidentiality not protected	0.657						0.645			
Promotes discrimination against groups of people	0.759						0.697			
Leads to unforeseen consequences	0.697						0.682			
Affects my employability	0.765						0.818			
Renders me unable to get insured	0.724						0.760			
Can be misused by corporate or government bodies	0.749						0.735			
*Attitudes towards genetics*										
Will help people to live better lives			0.748						0.686	
Valuable for early detection of diseases			0.625						0.746	
Will help people to live longer and better			0.739						0.741	
Can help a child to live a better life			0.653						0.726	
% of explained variance	17.551	15.439	9.438	7.853	6.366	31.584	11.637	9.747	7.036	5.850
Initial Eigenvalues	3.686	3.242	1.982	1.649	1.337	6.633	2.444	2.047	1.478	1.228
Cronbach alpha	0.821	0.759	0.688	0.676	0.701	0.890	0.835	0.790	0.764	0.734
Item-scale correlations	0.519–0.638	0.445–0.610	0.394–0.585	0.422–0.491	0.542	0.645–0814	0.488–0.717	0.525–0.603	0.526–0.594	0.581
CR	0.87	0.83	0.79	0.77	0.83	0.89	0.87	0.84	0.82	0.86
AVE	0.53	0.50	0.48	0.46	0.71	0.62	0.53	0.58	0.53	0.75

The questionnaire items were grouped in an expected and similar way for both samples, and it was revealed that the examined factors affect students’ intention to adopt PGx testing. The first two factors, in terms of percentage of explained variance, in both samples refer to the benefits of PGx on drug management and the concerns associated with the use of genetic testing. The next two factors were related to student attitudes towards genetics and their benefits on disease management, while the fifth factor was about students’ self-evaluation of their genetic training.

### Comparative analysis of students’ views on questionnaire items

Students’ answers from both groups were compared using the nonparametric Mann–Whitney test (Table 3). It is shown that Greek students are more confident about the contribution of genetic testing in terms of disease diagnosis, prevention and prognosis, whereas Malay students are more convinced about PGx benefits on drug management, with respect to the reduction in healthcare expenses on medication, the potential decrease in adverse drug reactions’s incidence, in symptoms’ severity, and in the number of exacerbations. However, students from both countries appear to be particularly convinced about the benefits of genetic testing on disease management (mean values ranged from 4.07 to 4.35), while Greek students felt to be less adequately trained to discuss with a family member the results of a genetic test and consult him/her accordingly. The mean values for the relevant statements range from 3.73 to 3.95 for the Malay sample, and from 3.20 to 3.77 for the Greek sample.

**Table 3:** Comparison of students’ views on questionnaire items

Factors and variables	Greece (*N* = 205)		Malaysia (*N* = 201)		Mann–Whitney test	
	Mean	SD	Mean	SD	Z	Asymp. sig. (2-tailed)
*Genetics training*						
Draw a pedigree	3.35	1.30	3.27	0.99	− 1.280	0.201
Discuss with a family the results of a genetic test and consult	2.56	1.20	3.13	1.05	− 4.939	0.000
*Benefits on disease management*						
Diagnosis	4.35	0.92	4.09	0.72	− 4.697	0.000
Treatments	4.13	0.95	4.07	0.76	− 1.695	0.090
Prevention	4.26	0.89	4.10	0.77	− 2.718	0.007
Prognosis	4.25	0.90	4.09	0.80	− 2.713	0.007
*Benefits on drug management*						
Drug efficacy increase	3.77	0.99	3.95	0.75	− 1.520	0.129
Medication cost reduction	3.20	1.03	3.73	0.89	− 5.495	0.000
Incidence of adverse drug reactions reduction	3.55	0.95	3.89	0.76	− 3.580	0.000
Severity of adverse drug reactions reduction	3.53	0.92	3.82	0.83	− 2.943	0.003
Exacerbation reduction	3.37	0.97	3.70	0.83	− 3.213	0.001
*Concerns (risk) about genetics*						
Privacy and confidentiality not protected	2.72	1.17	3.40	1.02	− 5.922	0.000
Promotes discrimination against groups of people	2.89	1.27	3.58	0.94	− 5.561	0.000
Leads to unforeseen consequences	3.56	1.11	3.66	0.90	− 0.410	0.682
Affects my employability	3.62	1.16	3.69	0.90	− 0.194	0.846
Renders me unable to get insured	3.36	1.18	3.70	0.88	− 2.706	0.007
Can be misused by corporate or government bodies	3.78	0.99	3.78	0.91	− 0.540	0.589
*Attitudes towards genetics*						
Will help people to live better lives	4.54	0.66	4.28	0.78	− 3.562	0.000
Valuable for early detection of diseases	4.61	0.76	4.31	0.81	− 4.840	0.000
Will help people to live longer and better	4.35	0.81	4.01	0.87	− 4.208	0.000
Can help a child to live a better life	4.18	0.94	4.05	0.78	− 2.423	0.015
*Intention to adopt genetics for self-use*						
To know my own genetic profile	4.18	1.03	4.31	0.93	− 1.039	0.299
To know my potential future diseases	4.07	1.12	4.20	0.93	− 0.622	0.534

Moreover, it is noticed that for both groups (mean 3.78) the potential misuse of genetic information by corporate or governmental bodies is the most important concern, followed by the possible impact to employability and fears about unforeseen consequences due to the widespread use of genetic research (means varied from 3.56 to 3.69). Greek students appear to be considerably less worried than Malay students about the potential discrimination and privacy issues, the promotion of discrimination against groups of people and the potential inability to get insured. In line with participants’ beliefs about the benefits of genetic testing on drug and disease management, students from both countries expressed positive attitudes towards genetic research and testing, especially in terms of its valuable contribution to the early detection of diseases (means > 4.5 for Greeks and ~ 4.3 for Malays). Nevertheless, Greek students are shown to be more enthusiastic about genetic research and testing outcomes compared to their counterparts.

Despite the noteworthy differences between the two groups’ perceptions regarding the aforementioned factors, students from both countries exhibited almost similar favourable intentions to adopt genetic testing for personal use, with mean values varying from 4.1 to 4.3 (Table 3). This trend provides an additional impetus to conduct the comparative path analysis.

### Path analysis

**Fig. 2:**
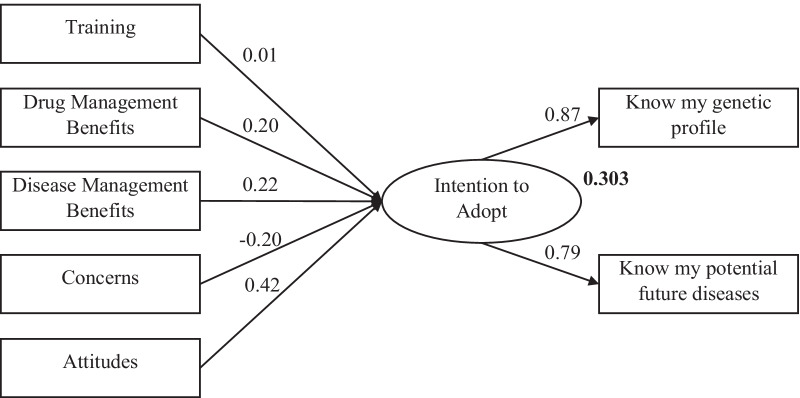
Path diagram of Greek sample (standardized estimates)

**Fig. 3:**
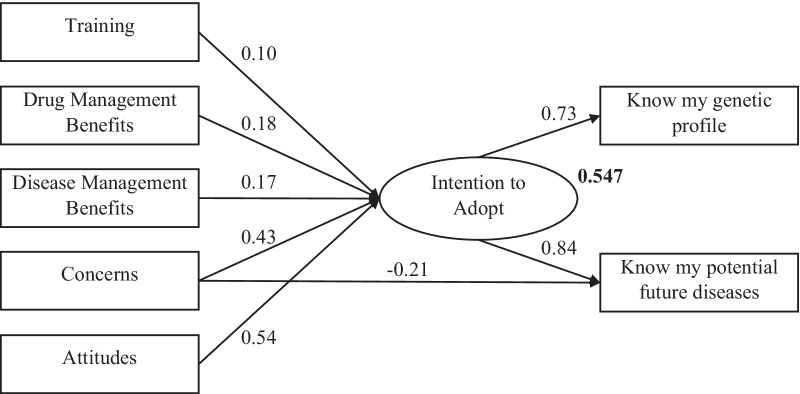
Path diagram of Malaysian sample (standardized estimates)

Structural equation modelling (SEM) and, in particular, path analysis were conducted to estimate the impact of the revealed factors to students’ intention to adopt genetic testing applications (Figs. 2, 3). Anderson–Rubin factor scores produced by the exploratory factor analysis for five selected predicting factors of students’ intention to adopt genetic testing were applied in this analysis. Model fitness indices (Table 4) unveil that both SEM models are good fit [27, 28].

**Table 4:** Model fitness indices for both SEM models

Index	Greece	Malaysia	Accepted value(s)*
CMIN/DF	0.568	0.331	2.0–5.0
*p*	0.892	0.987	≥ 0.05
IFI	1.033	1.055	≥ 0.95
NFI	0.96	0.975	≥ 0.95
TLI	1.051	1.094	≥ 0.95
CFI	1.000	1.000	≥ 0.95
RMSEA	0.000	0.000	≤ 0.08
PCLOSE	0.986	0.999	≥ 0.05

^*^Sources Hooper et al. [27], Schreiber et al.[28]

Path diagram (Fig. 1), regression weights (Additional file 1: Table S2) and squared multiple correlations (*R*^2^) of Intention to Adopt (0.303) for the Greek sample reveal that all examined factors, except of training, have a significant impact on students’ intention to adopt genetic testing for personal use. However, there is an observed variation on the factors’ regression coefficients. Attitudes as a factor exert the most significant positive effect on students’ intention (0.416) compared to the rest of the factors. Standardized regression results concerning the variable of perceived benefits on disease and drug management are around 0.20–0.22, indicating a positive impact on students’ intention. On the contrary, concerns exert a negative impact (− 0.196) towards the testing adoption, as expected. Consequently, these four factors’ influence (directly or not) students’ intention and its antecedents (variables) (“*I want to know my own genetic profile”* and “*I want to know what kind of diseases I could get in the future”*) (Additional file 1: Table S3). Path analysis including Greek students’ demographics reveals that study year was their only characteristic with a significant impact on their intention to adopt genetic testing, raising the model’s *R*^2^ to 0.342. In particular, the negative regression weight (− 0.21) indicates that 1st-year students were more inclined to adopt a genetic test compared to their 2nd-year peers (Additional file 1: Fig. S1, Table S6).

SEM analysis for the Malaysian sample demonstrates similar findings to those of the Greek sample (Fig. 3, Additional file 1: Tables S4 and S5). Indeed, training constitutes the only factor found not to influence students’ intention towards genetic testing adoption, while the other four factors have a significant effect. Attitudes’ factor impinges more on students’ intention, with its standardized regression estimated to be considerably higher (0.539) compared to the Greek sample (0.416). Rather interestingly, concerns about genetics have a noteworthy positive impact (0.431) on students’ intentions, contrary to the expected relationship and the observed in the Greek sample (− 0.196). This fact implies that the more the Malay students are concerned about the genetics risks, the more are inclined to adopt genetic testing. This ostensible paradox may indicate that high level of genetic knowledge is positively associated with both students’ worries and intention to adopt genetic testing. Yet, it is worth mentioning that the concerns as a factor is proven to have a relatively modest negative impact (− 0.215) on students’ intentions to know what kind of diseases they could get in the future. The last two factors, genomics benefits on disease and drug management, appear to exert a rather positive impact (0.17 and 0.18, respectively) on students’ intentions like the Greek sample (0.22 and 0.20). The squared multiple correlation (*R*^2^) of students’ intention to adopt genetic testing was much higher (0.547) than the observed in the Greek sample (0.303). This may be due to the composition of the two samples, as the Malay participants are expected to be more knowledgeable and familiar with the corresponding topics than their Greek counterparts. Path analysis including Malay students’ demographics manifests that the examined characteristics exert almost a null impact on their intention to genetic test adoption (Additional file 1: Fig. S2, Table S7).

## Discussion

Genomics and genetic testing applications have significantly changed the way modern clinical routine works. The wide range of testing applications is believed to improve the whole workflow of drug and disease management, on the grounds that it can enhance disease diagnosis, offer personalised therapeutic schemes, reduce the time and money required for treatment, increase a patient’s life expectancy and ameliorate a person's quality of life during therapy [1]. Although it is proven that genetic testing has a positive impact on clinical practice, its adoption rate remains low across the different healthcare systems due to social, educational, religious, cultural and legal barriers as many studies have previously shown [16, 29].

Even if there is a great number of publications addressing the possible barriers that may impede PM implementation and the use of different types of genetic testing, there are only a few studies focusing on determining and empirically investigating the factors and their association with the testing adoption. According to Salleh et al. [6], exploring a person’s behaviour, action and attitude could predict a positive relationship between a person’s intention and his tendency to adopt a new technology [7].

For instance, Mustapa et al. [25] have investigated the intention to adopt PGx, a type of genetic testing, using TAM among Malaysian stakeholders (healthcare professionals, public, policymakers) and they concluded in similar results to other TAM studies [26]. More precisely, they showed that perceived benefits and risks of PGx along with concerns, self-efficacy and level of knowledge affected the public's willingness to adopt PGx. Chen et al. [26] had conducted a similar research in Taiwanese physicians to investigate the professionals’ attitudes and their intention to adopt genomic medicine, and they concluded that education level and knowledge related to genomics is positively associated with physicians intention to adopt genomic medicine in their practice along with other external factors such as the existing health policies, administration support, cultural setting and other important elements related to the feasibility of this innovation in everyday clinical practice [27]. The above studies are focusing on healthcare professionals and not on the health science students which are the future generations of professionals. Our study is the first that examines the impact of selected factors on health science students’ intention to adopt genetic testing using TAM, while it compares two different cultural groups: a European, (Greece), and an Asian, (Malaysia), with the aim to highlight any existing similarities and differences between them.

Based on our results, it was illustrated that students from both groups appear to be particularly convinced about the benefits of genetic testing on disease management thanks to its contribution in the disease prognosis and on the improvement of patient’s quality of life, a finding that is also supported by Mahmutovic et al. [17]. More precisely, Malay students tend to believe more in the significance of genetic testing applications, such as PGx, in drug management than in disease management compared to their Greek counterparts. This observation may have a double meaning.

On the one hand, it might be related to the fact that the majority of Greek respondents were in their first- or second-year of studies and thus, they were not aware of the impact of drug management on patient’s overall management. According to Yau and Haque [30], it was indicated that the knowledge of PGx among students varied depending on the course of study and age, while Malay students had the same results in relevant questions, a fact that underlies the reliability and reproducibility of our observations [31]. On the other hand, it could be a result of cultural difference since pharmacists in Malaysia have a broader participation in the complete control of medication supply in comparison with their colleagues in Greece [31]. Indeed, Malay pharmacists have the authority to do medication reviews, identify medication issues and provide recommendations for better drug administration to patients and doctors.

Furthermore, the major students’ concern was the security of their genetic information and their potential misuse by corporate or government bodies. Malay students were shown to be extremely worried about it along with the existence of phenomena of racial and religious discrimination and privacy deprivation. Cheung et al. [32] stated that the majority of undergraduate students believed that the biggest threats of genetic testing, implementation were “Patient Privacy” (80%) and “Data Confidentiality” (68%), supporting our findings. In general, many studies have pointed out similar conclusions especially in terms of religiosity and religious restrictions [33, 34]. However, in general, Asians were found to worry more about the improper use of their data in many sectors of their lives compared to Western societies [6].

The noticeable difference between Malay and Greek students could also be explained by the existence and implementation of strict European directives and legislations such as GDPR [35]. Greece has complied with these laws and citizens’ personal data are legally protected, while there is not a similar legal framework in Malaysia. In addition to, as Mitropoulou et al., [36] have mentioned, Greek stakeholders in the healthcare sector and especially policymakers had a positive attitude towards genetic testing and they were willing to overcome any potential barrier to enhance PM implementation along with genetic testing.

Another noteworthy observation is related to the positive attitude of students from both groups towards the genetic testing for personal purposes to find out their genetic makeup and their prevalence for disease. In the literature, there is evidence that a small number of healthcare professionals claimed to have ordered or performed a genetic testing for themselves in the last six months. For example, 14.7% of respondents in Bank et al. [37] study reported having ordered or recommended genetic testing like PGx in the last 6 months, similar to 12.7% of respondents in Stanek et al. [38]. However, it seems that undergraduate students (83%) are more prone to undergo genetic testing to find out their genetic profile in the future [39]. Mahmutovic et al. [17] highlighted that 40% of participants were willing to undertake a genetic test in the future, while Sindi et al. [20] showed that 80.5% of students wanted to take a genetic test for themselves [17, 21].

Finally, training was also a significant concern for both groups, an observation that is really common in the literature [18]. Greek students felt to be less adequately trained to interpret the genetic data of their family members and discuss the results with them in order to consult them, whereas their Malay counterparts were more confident with that aspect. This difference may be attributed to the fact that almost 84% of Greek participants attended the first year of their studies and they lacked experience, while 92% of the Malay sample were in their second and third year and, hence, they had already attended more courses relevant to human genomics, genetic testing and PM to gain some experience.

However, based on the path analysis results, it seems that training constituted the only factor which did not influence students’ intentions towards genetic testing adoption, while attitude is the most influential factor with rather high impact followed by genetic testing benefits. This fact is in accordance with the fact that Malay health science students are still rather worried about the potential perils associated with genetic testing even if they are highly convinced about the benefits of testing on both drug and disease management. This paradox indicates that a high level of genetic knowledge may be potentially positively associated with both students’ worries towards and intentions to adopt genetic testing.

The study results provide crucial evidence of the proposed model’s external validity, although, in general, external validity’s evaluation always poses significant challenges. The questionnaire survey has been conducted in two quite different cultures, and the actual correlation coefficients between the predicting factors and students’ intentions to adopt genetic testing were rather similar in these two cultural settings.

This study has a few limitations related to the composition of the sample. In particular, the Greek sample consisted mainly of first year students (around 84%) with the rest of cohort attending the second year, while the Malay sample consisted of older students. This discrepancy may explain the better statistical results (exploratory factor analysis, path analysis) of the Malay sample compared to the Greek one and affect the correlations. Moreover, the samples from both countries derived from students attending one public University accordingly.

## Conclusions

This study shows that TAM has been successfully applied in many studies to explain the acceptance and the intention to adopt several health innovations. By using an extended version of TAM adapted for the needs of these research questions, and a series of multivariate statistical analyses, a new reliable and promising model has been developed to interpret health science students’ intentions to adopt genetic testing such as PGx in different cultural settings, with good fit thanks to the results of validity and reliability tests along with the observed similarities of the two samples.

It was shown that the intention of health science students to adopt PGx is affected by several factors such as attitudes, the perceived benefits of genetic testing, the risks and concerns of such technology. Our study model is suitable to investigate the factors influencing the intention to adopt genetic testing services even in different cultural settings. In the future, this model could have further marketing implications in different domains of the healthcare sector which will improve the adoption rate of genetic testing. For example, it would be used to upgrade Universities’ curriculum to assess the perception of students and then incorporate students’ programs accordingly. In parallel, by enacting strict and universal legislations for the protection of sensitive personal data such as the genomic data, it is possible to overcome some of the main barriers and convince the future healthcare professionals to widely implement PGx in the clinical setting. Finally, another marketing application is the extension of our model applicability in other healthcare stakeholders such as patients, public, policymakers to understand in depth the intention to adopt PGx with the aim of improving and strengthening the presence of PGx in clinical routine and drug administration.

## Supplementary Information

**Additional file 1:** Survey questions and extensive explanation of path analysis results for both groups. The file includes the questions of the survey, demonstration of regression weights as derived upon SEM analysis. and the effects of each factor in students’ intention to adopt genetic testing. Also, it presents more detailed path diagrams of all examined factors in figures  for both groups.

## Data Availability

Please contact author for data requests.

## Abbreviations

PM: Personalised medicine
PGx: Pharmacogenomics
TAM: Technology acceptance model
PU: Perceived usefulness
PEU: Perceived ease of use
Std Dev: Standard deviation
AVE: Average variance explained
CR: Composite reliability
FA: Factor analysis
KMO: Kaiser–Meyer–Olkin
SEM: Structural equation modelling
CMIN/DF: Chi-square fit statistics/degree of freedom
IFI: Incremental fit index
NFI: Normed fit index
TLI: Tucker–Lewis index
CFI: Comparative fit index
RMSEA: Root mean squared error approximation
PCLOSE: *p* Value of the null hypothesis that the RMSEA equals 0.05 (a close-fitting model)
