# Supplementary material for: Examining key factors impact on health science students’ intentions to adopt genetic and pharmacogenomics testing: a comparative path analysis in two different healthcare settings
Source: Hum Genomics. 2022 Mar 14;16:9. doi: 10.1186/s40246-022-00382-3 (PMC8919586; doi:10.1186/s40246-022-00382-3)
Supplement: Supplementary file 1 — Additional file 1: Survey questions and extensive explanation of path analysis results for both groups. The file includes the questions of the survey, demonstration of regression weights as derived upon SEM analysis. and the effects of each factor in students’ intention to adopt genetic testing. Also, it presents more detailed path diagrams of all examined factors in figures for both groups. [file 40246_2022_382_MOESM1_ESM.docx]

**Supplementary Material**

**Examining key factors impact on health science students’ intentions to adopt genetic and pharmacogenomics testing: A comparative path analysis in two different healthcare settings**

**Margarita-Ioanna Koufaki ^1^, Stavroula Siamoglou ^1^ George P. Patrinos ^1,2,3^, Konstantinos Vasileiou ^1^**

^1^ University of Patras School of Health Sciences, Department of Pharmacy, Laboratory of Pharmacogenomics and Individualized Therapy, Patras, Greece

^2^ United Arab Emirates University, College of Medicine and Health Sciences, Department of Pathology, Al-Ain, United Arab Emirates

^3^ United Arab Emirates University, Zayed Center for Health Sciences, Al-Ain, United Arab Emirates

**SUPPLEMENTAL TABLE 1.** Factors and Variables included in the Survey Instrument

| *Genetics training* | |
| --- | --- |
| TRN1 | I feel adequately trained with regard to draw a pedigree |
| TRN2 | I feel adequately trained to discuss with a family the results of a genetic test and consult them accordingly. |
| *Genomics Benefits on Disease Management* | |
| DSM1 | Genomic knowledge is helpful for: disease diagnosis |
| DSM2 | Genomic knowledge is helpful for: disease treatments |
| DSM3 | Genomic knowledge is helpful for: disease prevention |
| DSM4 | Genomic knowledge is helpful for: disease prognosis |
| *Pharmacogenomics Benefits on Drug Management* | |
| DRM1 | I believe that pharmacogenomics: increases drug efficacy |
| DRM2 | I believe that pharmacogenomics: reduces healthcare cost of medication by rationalizing drug use |
| DRM3 | I believe that pharmacogenomics: reduces the incidence of adverse drug reactions |
| DRM4 | I believe that pharmacogenomics: reduces the severity of adverse drug reactions |
| DRM5 | I believe that pharmacogenomics: reduces the number of exacerbation |
| *Concerns about Genetics* | |
| CON1 | I am worried that: my privacy and confidentiality is not protected if performed a genetic test |
| CON2 | I am worried that: the findings from genetic research promotes discrimination against groups of people |
| CON3 | I am worried that: the widespread use of genetic research may lead to unforeseen consequences |
| CON4 | I am worried that: the result of my genetics testing will affect my employability if I have a serious genetic defect that will be made known to my employer |
| CON5 | I am worried that: the result of genetics testing may render me unable to get insured, due to serious genetic defect. |
| CON6 | I am worried that: genetic information obtained can be misused by corporate or government bodies |
| *Attitudes towards Genetics* | |
| ATT1 | I believe that findings from genetic research will help people to live better lives |
| ATT2 | I believe that genetics testing for early detection of diseases is valuable |
| ATT3 | I think that knowledge about genetic background of disease will help people to live longer and better |
| ATT4 | I believe that genetic research can help a child to live a better life |
| *Intentions to Adopt Genetics for Self Use* | |
| INT1 | I want to know my own genetic profile |
| INT2 | I want to know what kind of diseases I could get in the future |

**SUPPLEMENTAL TABLE 2.** Regression Weights for the Greek sample

|  |  |  | Standardized estimate | S.E. | C.R. | P |
| --- | --- | --- | --- | --- | --- | --- |
| Intention to Adopt | 🡨 | Training | .006 | .061 | .085 | ,932 |
| Intention to Adopt | 🡨 | Drug Management Benefits | .204 | .066 | 2.771 | ,006 |
| Intention to Adopt | 🡨 | Disease Management Benefits | .222 | .060 | 3.273 | ,001 |
| Intention to Adopt | 🡨 | Concerns | -.196 | .064 | -2.710 | ,007 |
| Intention to Adopt | 🡨 | Attitudes | .416 | .063 | 5.827 | *** |
| Know my genetic profile | 🡨 | Intention to Adopt | .870 | .130 | 7.761 | *** |
| Know my potential future diseases | 🡨 | Intention to Adopt | .795 |  |  |  |

***: p< 0.001

**SUPPLEMENTAL TABLE 3.** Standardized direct, indirect and total effects for the Greek sample

|  |  | Attitudes | Concerns | Disease Management Benefits | Drug Management Benefits | Training | Intention to Adopt |
| --- | --- | --- | --- | --- | --- | --- | --- |
|  | Intention to Adopt | .416 | -.196 | .222 | .204 | .006 | .000 |
| Total | Know my genetic profile | .331 | -.156 | .177 | .162 | .005 | .795 |
|  | Know my potential future diseases | .362 | -.170 | .193 | .178 | .005 | .870 |
|  | Intention to Adopt | .416 | -.196 | .222 | .204 | .006 | .000 |
| Direct | Know my genetic profile | .000 | .000 | .000 | .000 | .000 | .795 |
|  | Know my potential future diseases | .000 | .000 | .000 | .000 | .000 | .870 |
|  | Intention to Adopt | .000 | .000 | .000 | .000 | .000 | .000 |
| Indirect | Know my genetic profile | .331 | -.156 | .177 | .162 | .005 | .000 |
|  | Know my potential future diseases | .362 | -.170 | .193 | .178 | .005 | .000 |

**SUPPLEMENTAL TABLE 4.** Regression Weights for the Malaysian sample

|  |  |  | Standardized estimate | S.E. | C.R. | P |
| --- | --- | --- | --- | --- | --- | --- |
| Intention to Adopt | 🡨 | Training | .103 | .050 | 1.624 | .104 |
| Intention to Adopt | 🡨 | Drug Management Benefits | .180 | .050 | 2.819 | .005 |
| Intention to Adopt | 🡨 | Disease Management Benefits | .166 | .050 | 2.600 | .009 |
| Intention to Adopt | 🡨 | Concerns | .431 | .080 | 4.204 | *** |
| Intention to Adopt | 🡨 | Attitudes | .539 | .057 | 7.391 | *** |
| Know my genetic profile | 🡨 | Intention to Adopt | .732 | .126 | 6.892 | *** |
| Know my potential future diseases | 🡨 | Intention to Adopt | .839 |  |  |  |
| Know my potential future diseases | 🡨 | Concerns | -.215 | .083 | -2.427 | .015 |

***: p< 0.001

**SUPPLEMENTAL TABLE 5.** Standardized direct, indirect and total effects for the Malaysian sample

|  |  | Attitudes | Concerns | Disease Management Benefits | Drug Management Benefits | Training | Intention to Adopt |
| --- | --- | --- | --- | --- | --- | --- | --- |
|  | Intention to Adopt | .539 | .431 | .166 | .180 | .103 | .000 |
| Total | Know my genetic profile | .452 | .147 | .139 | .151 | .087 | .839 |
|  | Know my potential future diseases | .394 | .316 | .121 | .132 | .076 | .732 |
|  | Intention to Adopt | .539 | .431 | .166 | .180 | .103 | .000 |
| Direct | Know my genetic profile | .000 | -.215 | .000 | .000 | .000 | .839 |
|  | Know my potential future diseases | .000 | .000 | .000 | .000 | .000 | .732 |
|  | Intention to Adopt | .000 | .000 | .000 | .000 | .000 | .000 |
| Indirect | Know my genetic profile | .452 | .362 | .139 | .151 | .087 | .000 |
|  | Know my potential future diseases | .394 | .316 | .121 | .132 | .076 | .000 |

**SUPPLEMENTAL TABLE 6.** Regression Weights for the Greek sample including demographics

|  |  |  | Standardized estimate | S.E. | C.R. | P |
| --- | --- | --- | --- | --- | --- | --- |
| Intention to Adopt | 🡨 | Training | .001 | .060 | .016 | .987 |
| Intention to Adopt | 🡨 | Drug Management Benefits | .216 | .065 | 2.985 | .003 |
| Intention to Adopt | 🡨 | Disease Management Benefits | .219 | .059 | 3.308 | *** |
| Intention to Adopt | 🡨 | Concerns | -.225 | .064 | -3.139 | .002 |
| Intention to Adopt | 🡨 | Attitudes | .395 | .062 | 5.677 | *** |
| Intention to Adopt | 🡨 | Department | .086 | .129 | 1.236 | .216 |
| Intention to Adopt | 🡨 | Gender | .013 | .124 | .197 | .844 |
| Intention to Adopt | 🡨 | Study Year | -.211 | .158 | -2.883 | .004 |
| Know my genetic profile | 🡨 | Intention to Adopt | .861 | .118 | 8.348 | *** |
| Know my potential future diseases | 🡨 | Intention to Adopt | .803 |  |  |  |

***: p< 0.001

**SUPPLEMENTAL TABLE 7.** Regression Weights for the Malaysian sample including demographics

|  |  |  | Standardized estimate | S.E. | C.R. | P |
| --- | --- | --- | --- | --- | --- | --- |
| Intention to Adopt | 🡨 | Training | .105 | .050 | 1.667 | .096 |
| Intention to Adopt | 🡨 | Drug Management Benefits | .180 | .050 | 2.819 | .005 |
| Intention to Adopt | 🡨 | Disease Management Benefits | .168 | .050 | 2.660 | .008 |
| Intention to Adopt | 🡨 | Concerns | .434 | .081 | 4.216 | *** |
| Intention to Adopt | 🡨 | Attitudes | .538 | .057 | 7.505 | *** |
| Intention to Adopt | 🡨 | Department | .042 | .105 | .642 | .521 |
| Intention to Adopt | 🡨 | Gender | .035 | .115 | .533 | .594 |
| Intention to Adopt | 🡨 | Study Year | -.045 | .073 | -.699 | .484 |
| Know my genetic profile | 🡨 | Intention to Adopt | .729 | .125 | 6.862 | *** |
| Know my potential future diseases | 🡨 | Intention to Adopt | .846 |  |  |  |
| Know my potential future diseases | 🡨 | Concerns | -.219 | .084 | -2.453 | .014 |

***: p< 0.001

**SUPPLEMENTAL FIGURE 1.** Path diagram of the Greek sample including demographics

Attitudes

Concerns

Disease Management Benefits

Drug Management Benefits

Training

Know my genetic profile

Know my potential future diseases

0.00

0.22

0.22

-0.22

0.39

0.86

0.80

**0.342**

Department

Study Year

Gender

-0.21

0.01

0.09

**SUPPLEMENTAL FIGURE 2.** Path diagram of the Malaysian sample including demographics

Attitudes

Concerns

Disease Management Benefits

Drug Management Benefits

Training

Know my genetic profile

Know my potential future diseases

0.11

0.18

0.17

-0.43

0.54

0.73

0.85

**0.552**

Department

Study Year

Gender

-0.05

0.03

0.04

-0.22
